# Breath-Hold Diving-Related Decompression Sickness with Brain Involvement: From Neuroimaging to Pathophysiology

**DOI:** 10.3390/tomography8030096

**Published:** 2022-04-19

**Authors:** José Manuel Sánchez-Villalobos, María Lorenza Fortuna-Alcaraz, Laura Serrano-Velasco, Ángel Pujante-Escudero, Carmen María Garnés-Sánchez, Jorge Edverto Pérez-Garcilazo, Agustín Olea-González, José Antonio Pérez-Vicente

**Affiliations:** 1 Department of Neurology, University Hospital Complex of Cartagena (Santa Lucia University Hospital—Santa María del Rosell University Hospital), 30202 Cartagena, Spain; loforta@yahoo.es (M.L.F.-A.); cmaria.garnes@gmail.com (C.M.G.-S.); japevik@gmail.com (J.A.P.-V.); 2 Department of Cell Biology and Histology, Faculty of Medicine/Dentistry, Biomedical Research Institute of Murcia (IMIB-Arrixaca), University of Murcia, 30100 Murcia, Spain; 3 Department of Radiology, University Hospital Complex of Cartagena (Santa Lucia University Hospital—Santa María del Rosell University Hospital), 30202 Cartagena, Spain; laurasevel@hotmail.com; 4 Department of Underwater and Hyperbaric Medicine, Spanish Navy Diving Center, La Algameca Naval Station, 30205 Cartagena, Spain; apapes77@gmail.com (Á.P.-E.); perezgarcilazo@outlook.es (J.E.P.-G.); aoleag@fn.mde.es (A.O.-G.)

**Keywords:** decompression sickness, breath-hold diving, magnetic resonance imaging, diffusion-weighted images, apparent diffusion coefficient maps, PRES

## Abstract

Central nervous system involvement related to decompression sickness (DCS) is a very rare complication of breath-hold diving. So far, it has been postulated that repeated dives with short surface intervals represent a key factor in the development of breath-holding-related DCS. We report the case of a breath-hold diver who, after repeated immersion, developed DCS with brain involvement. After treatment in a hyperbaric chamber, there was a clinical improvement in the symptoms. Magnetic resonance imaging of the brain showed hyperintense lesions in long-time repetition sequences (FLAIR, T2WI) in the left frontal and right temporal lobes. Diffusion-weighted imaging (DWI) sequences and the apparent diffusion coefficient (ADC) map were characteristic of vasogenic edema, allowing us to exclude the ischemic nature of the process. These findings, together with the acute clinical presentation, the resolution of lesions in evolutionary radiological controls and the possible involvement of blood–brain barrier/endothelial dysfunction in DCS, could suggest a new form of posterior reversible encephalopathy syndrome (PRES)-like presentation of DCS. This would represent a novel mechanism to explain the pathophysiology of this entity. We conducted a literature review, analyzing the pathophysiological and neuroimaging characteristics of DCS in breath-hold diving based on a case of this rare disease.

## 1. Introduction

Diving has become a widespread practice in the world today, encompassing a wide range of activities from scuba diving (as a sport or professionally) and snorkeling to breath-hold diving. Breath-hold diving is mostly practiced at shallow depths and for short periods of time by the general public; in contrast, spearfishermen or competitive breath-hold divers may make repeated dives to depths of more than 30 m and for longer periods of time (>1 min) [1,2]. One of the most serious complications of both breath-hold diving and scuba diving is decompression sickness (DCS). In general, DCS is caused by the appearance of inert gas bubbles in the blood and/or tissues during or after a reduction in environmental pressure [3]; DCS can also affect aviators, astronauts and compressed air workers. Currently, DCS with neurological symptoms is estimated to occur in 2.7 out of 10,000 divers [3]. Although previous studies have been published attempting to correlate neuroradiological findings with the pathophysiology of neurological DCS, especially in breath-hold diving, this remains unclear at present [4,5,6,7].

Despite this, advances in modern neuroimaging techniques in recent years have allowed us to gain a deeper understanding of numerous neurological pathologies. This improvement has been partly due to the development of diffusion-weighted imaging (DWI) sequences and apparent diffusion coefficient (ADC) maps, which are very useful for a differential diagnosis between vasogenic and cytotoxic cerebral edema.

We report the case of a spearfishing breath-hold diver with brain DCS and conducted a review of the literature, analyzing the pathophysiological and neuroimaging features of DCS in breath-hold diving.

## 2. Case Report

A 31-year-old male came to the emergency department with symptoms of paresthesia and difficulty writing. There was no medical or surgical history of note, and he currently worked as an underwater fisherman.

The day before admission, while he was spearfishing, he began to have fatigue, as well as headache, paresthesia and weakness in the right hemibody. He also reported feeling “confused” and “disoriented”, even having difficulty getting out of the water. Finally, he managed to reach a dock and call for help. The summary of the patient’s dive profile consisted of a 3 h dive at a maximum depth of 30 m, with inter-immersion periods of less than 2 min on the surface and breath-hold times longer than two minutes. Two days earlier he had made another dive of approximately 4–5 h at a similar maximum depth (30 m) and with a similar dive profile.

**Figure 1:**
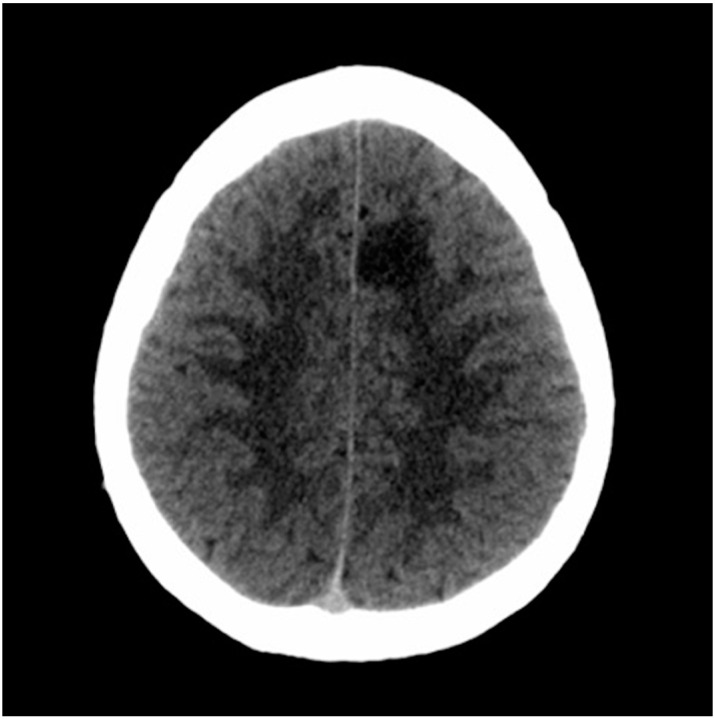
Computed tomography scan of the brain. The hypodense lesion in the medial left frontal lobe could be compatible, among other possibilities, with subacute ischemic stroke in the vascular territory of the left anterior cerebral artery.

After consulting the emergency room for these symptoms, he was discharged due to “muscle weakness after physical exercise”. As the hours passed, he reported an improvement in the weakness, although with persistent right brachiocrural paresthesia, as well as manipulative clumsiness of the right hand and a feeling of dullness in the head. Finally, given the persistence of symptoms, he decided to return to the emergency department and was then assessed by the on-call neurologist. The vital signs were blood pressure 128/68 mmHg, heart rate 82 bpm, oxygen saturation 100% and temperature 36.0 °C. Neurological examination showed the following: alert and oriented; language, speech and voice were preserved; normal cranial nerves; motor balance preserved; right Babinski’s sign; preserved exteroceptive sensitivity; coordination without dysmetria; and cautious gait, not ataxic. A computed tomography (CT) scan of the brain was urgently requested (Figure 1), showing findings compatible with a subacute ischemic lesion in the territory of the left anterior cerebral artery.

**Figure 2:**
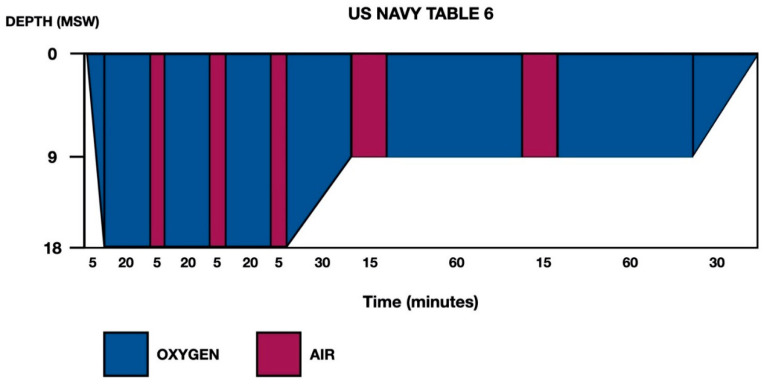
US Navy Treatment Table 6. Total elapsed time is 285 min, ascent time between stops (18–9 and 9–0) is 30 min (3 m/10 min) and maximum pressure is 18 m of seawater (msw).

Given the history of breath-hold diving with a risk profile for DCS, as well as the clinical-exploratory and neuroimaging findings, the patient was transferred to the reference hyperbaric chamber. Recompression was carried out in the hyperbaric chamber at 2.8 atmospheres absolute (ATA), and the patient presented progressive recovery of symptoms, for which treatment was completed using US Navy Table 6 (Figure 2). Abundant oral hydration was given, and the patient was recommended to remain near the hyperbaric chamber for 24 h. The patient was admitted to the Neurology Department (in the Stroke Unit, initially).

**Figure 3:**
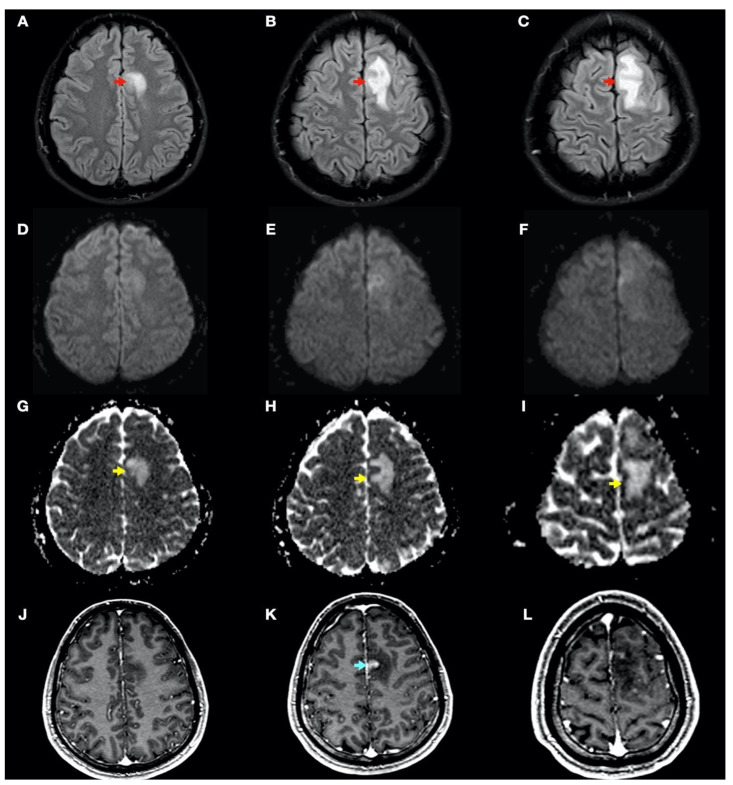
Brain magnetic resonance imaging (MRI): Images (**A–I**) from MRI 4 days after clinical onset; images (**J**–**L**) from MRI 5 days after clinical onset. Axial fluid-attenuated inversion recovery (FLAIR) images (**A**–**C**) show a left frontal cortico-subcortical hyperintense lesion (red arrows), also observed in other long-time repetition (TR) sequences (e.g., T2-weighted and proton density sequences). Axial diffusion-weighted imaging (DWI) (b1000) sequences (**D**–**F**) did not show a high signal intensity of lesions, whereas axial apparent diffusion coefficient maps (**G**–**I**) show a hyperintense lesion in this location (yellow arrows). (**J**–**L**) Axial gadolinium-enhanced 3D T1-weighted imaging (Gd T1WI) shows the presence of different areas of enhancement with irregular morphology (blue arrow), not suggestive of tumor enhancement.

**Figure 4:**
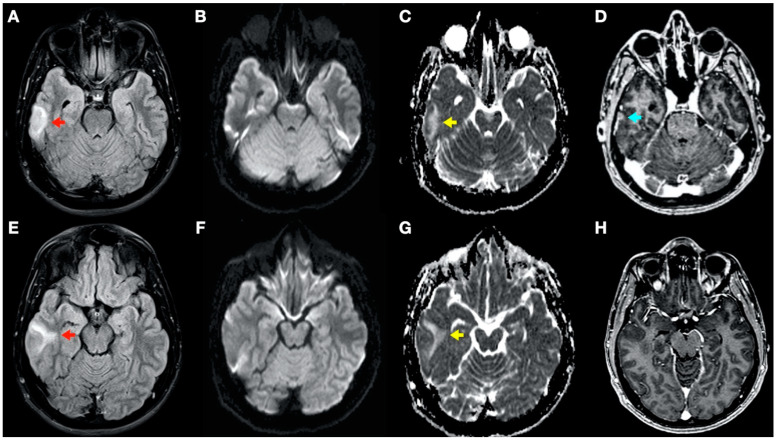
Brain magnetic resonance imaging (MRI): Images (**A**–**C**,**E**–**G**) from MRI 4 days after clinical onset; images (**D**,**H**) from MRI 5 days after clinical onset. Axial fluid-attenuated inversion recovery (FLAIR) images (**A**,**E**) show a right temporal cortico-subcortical hyperintense lesion with slight mass effect (red arrows). Axial diffusion-weighted imaging (DWI) sequences (**B**,**F**) did not show a high-signal intensity of lesions, whereas axial apparent diffusion coefficient maps (**C**,**G**), show a hyperintense lesion in this location (yellow arrows). (**D**,**H**) Axial gadolinium-enhanced 3D T1-weighted imaging (Gd T1WI) shows the presence of a slight enhancement of irregular morphology (blue arrow).

**Figure 5:**
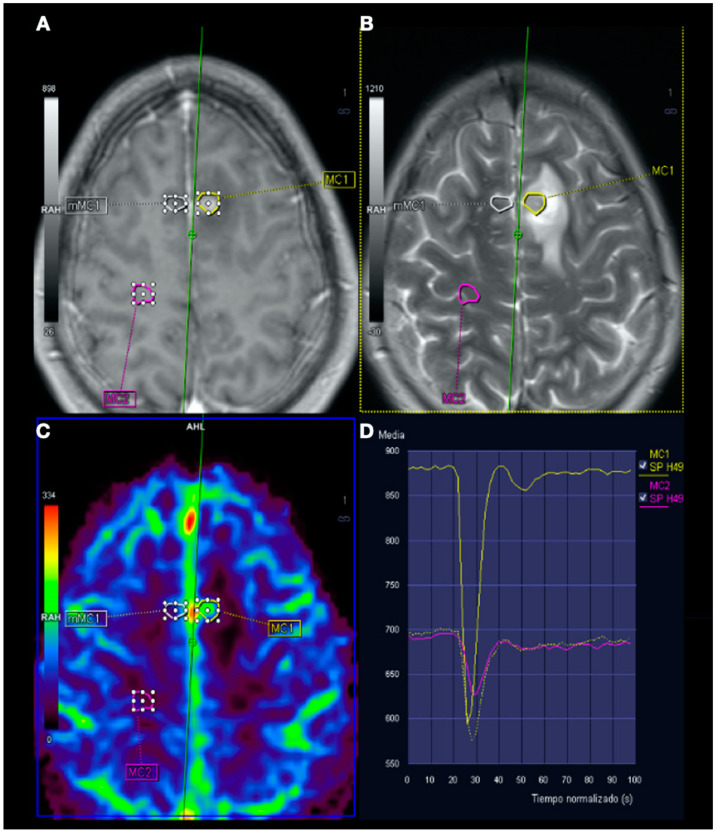
Magnetic resonance imaging (MRI) perfusion: Image (**A**), Gd T1WI; image (**B**), T2WI; image (**C**), MRI perfusion map corresponding to the corrected cerebral blood volume (CCBV); image (**D**), average curve. In the contrast enhancement area, there is an increase in cerebral blood volume (CBV) in relation to the contralateral side and normal subcortical white matter. In the average curves, the area under the curve is higher in the enhancement zone than in the other two zones.

During admission, the following complementary examinations were performed: brain magnetic resonance imaging (MRI; Figure 3, Figure 4 and Figure 5) showed right temporal and left frontal cortico-subcortical lesions, probably secondary to neurological involvement of DCS; neurosonological study of supra-aortic and intracranial arteries showed no atheromatosis or significant hemodynamic alterations; blood tests, including biochemistry, hemogram, coagulation study, serology and autoimmunity, were normal; transthoracic echocardiography was normal (including negative agitated saline contrast study for right-to-left shunt); and total body CT scan showed no evidence of neoplastic process.

After the extension study, given the lesions in the cerebral MRI suggestive of brain involvement due to DCS, the patient being asymptomatic from the clinical point of view and the absence of findings in the extension study that could explain the clinical picture, the patient was discharged from hospital with planned follow-up neurology consultations.

**Figure 6:**
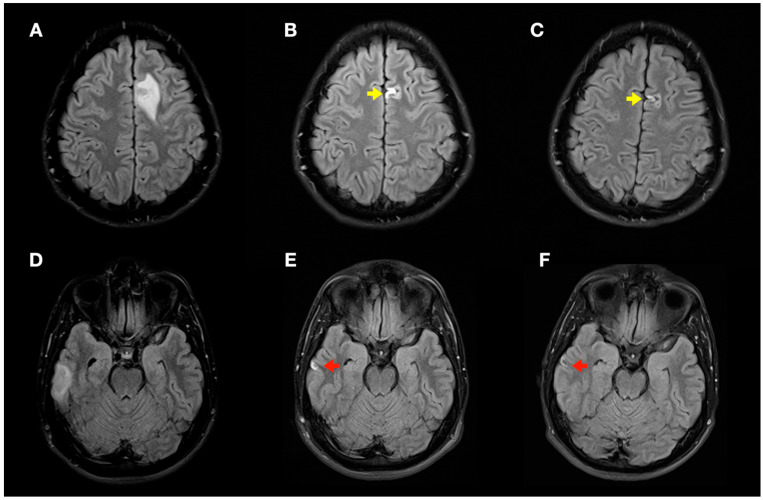
Radiological evolution of lesions secondary to decompression sickness: axial FLAIR images. (**A**,**D**) Initial images: left frontal cortico-subcortical hyperintense lesion (**A**) and right temporal cortico-subcortical hyperintense lesion (**D**). (**B**,**E**) Image control at 1 month: minimal hyperintense lesions in the right temporal (red arrow) and left frontal (yellow arrow) lobes of the brain. (**C**,**F**) Image control at 4 months: minimal chronic lesions compatible with cerebral malacia in the right temporal (red arrow) and left frontal (yellow arrow) lobes of the brain.

During follow-up, the patient did not present any new neurological symptoms, and radiological improvement was observed in successive neuroimaging tests (Figure 6), with only minimal chronic cortical lesions compatible with areas of cerebral malacia in the right temporal and left frontal lobes of the brain.

## 3. Discussion

Nowadays, the neurological involvement of DCS continues to be a serious problem related to diving. It predominantly affects the spinal cord, with brain involvement being less common [5], and even rarer in the case of breath-hold diving, which has made it difficult to obtain a more precise understanding of the pathophysiology of DCS.

Firstly, what we know about the pathophysiology of DCS could be summarized as follows: according to Henry’s law, at a constant temperature, the amount of gas that dissolves in a liquid depends on the solubility constant of each gas/liquid and the partial pressure of the gas. During diving, the ambient pressure increases with depth at a rate of 1 ATA (1013.25 hPa) every 10 m of the water column; therefore, during the dive, the partial pressure of the gases in the pulmonary alveolus increases, and an absorption gradient of these gases is established towards the pulmonary capillary, and from there to the rest of the organism; the oxygen is consumed in cellular metabolism, but this is not the case for the inert gas (nitrogen when diving with pressurized air), which accumulates in the diver. The amount of inert gas absorbed will depend on the depth and duration of the dive, and its distribution in the various organs and tissues will depend on their vascularization and composition (nitrogen dissolves more rapidly in fat tissues). On returning to the surface, the ambient pressure decreases and the inert gas gradient from the tissues to the lungs is reversed. If the ascent is too rapid, if the diver fails to make the needed decompression stops (decompression is omitted) or if there are several predisposing factors (physical exertion, obesity, hypothermia, hypobaric exposure after immersion, etc.), a state called *critical supersaturation* may be reached in which the inert gas cannot remain dissolved and bubbles appear in different locations. These bubbles may remain in the tissues or enter the venous system and may cause mechanical, biochemical or embolic damage, with a wide spectrum of manifestations [3,8]. A particular case that is much less frequent and less well known from a pathophysiological point of view is breath-hold diving-related DCS. In this scenario, breath-hold divers do not breathe pressurized air, and the only inert gas they take in is the nitrogen that remains in the lungs since the last breath before the dive. Thus, the DCS reported in the literature in pearl and Ama divers has been attributed to this progressive accumulation of residual nitrogen that increases with repetitive dives until it reaches the critical oversaturation that allows the formation of bubbles. It has been hypothesized that a key element in tissue nitrogen oversaturation would be repetitive dives with short inter-dive intervals [2].

Secondly, among the imaging techniques available for the study of neurological DCS, MRI represents the most accurate technique for the detection of pathological lesions in the spinal cord and brain [5]. To date, several MRI studies of neurological DCS have been published in the literature with different and often controversial results. These discrepancies could be explained by the lack of standardized neuroimaging protocols and the variability between the onset of clinical symptoms and the performance of the MRI study, as well as the high variability of different technical equipment [5]. Thus, although modern neuroimaging techniques have allowed us to deepen our understanding of the pathophysiology of DCS, the exact mechanism by which nitrogen bubbles cause damage to the nervous system is still widely debated. There are currently several theories supporting different pathophysiological mechanisms, most notably arterial occlusion, venous infarction and mechanical damage by autochthonous bubble formation. In brief, on the one hand, it has traditionally been considered that DCS with cerebral involvement would be due to the predominant involvement of the arterial circulation (stroke-like radiological imaging) [5,9], by paradoxical embolism in patients with patent foramen ovale (PFO) or by opening of pulmonary arterio-venous shunts in the case of large venous embolisms where the alveolar–capillary filter is exceeded [5,8]. Thus, previous studies have shown that the presence of a right-to-left shunt, as in the case of a PFO, significantly increases the risk of DCS in compressed-gas diving by facilitating a paradoxical pathway of arterial embolism [10,11,12,13]. This facilitating effect of the development of DCS, related to the right-to-left shunt, has also been suggested in some cases in breath-hold divers [14]. On the other hand, in DCS affecting the spinal cord, injury is thought to involve obstruction and congestion of venous drainage, in addition to direct injury by bubble formation within the cord itself [5,8,9,15]. Finally, in the case of neurological involvement of breath-hold diving-related DCS, knowledge of the pathophysiology is even less well understood, as this is a rarer pathology, with most published work on acute DCS in scuba divers and fewer reports on DCS in breath-hold diving [4,7,16,17,18,19].

Thirdly, regarding the treatment of DCS in breath-hold divers, although experience remains limited, it seems reasonable to adopt the same measures as those applied in the treatment of compressed-gas diving-related DCS. These would include immediate application of normobaric oxygen therapy at maximum fraction of inspired oxygen (FiO_2_), followed as soon as possible by recompression therapy in a hyperbaric chamber. The objective is to achieve an immediate reduction of the size of the inert gas bubbles formed (Boyle’s law), as well as to accelerate their redissolution (Henry’s law). In the case presented, US Navy Table 6 (Figure 2) was applied according to generally accepted protocols for DCS with neurological involvement [20,21].

In this complex scenario, we report an uncommon case of DCS in a spearfishing breath-hold diver. The diver had a high-risk dive profile, with repetitive breath-hold dives to a maximum depth of about 30 m for several hours and short surface intervals. He had a good response to hyperbaric chamber treatment (US Navy Table 6) and was clinically asymptomatic. Neuroimaging findings showed hyperintensity in long-TR sequences (FLAIR, T2WI), as well as in ADC mapping, and no hyperintensity in DWI sequences, compatible with the presence of vasogenic edema in the left frontal and right temporal lobes (Figure 3 and Figure 4). Regarding MRI perfusion, in the contrast enhancement area, there was an increase in cerebral blood volume (CBV) in relation to the contralateral side and normal subcortical white matter. In the average curves, the area under the curve was higher in the enhancement zone than in the other two zones, which would indicate an increase in perfusion (Figure 5). Initially, given the atypical nature of the lesions, with irregular enhancement in T1WI with intravenous contrast (although their radiological characteristics were not suggestive of neoplastic etiology) [22,23], an extension study with body CT was completed, showing no presence of neoplasia. Likewise, prior to the neuroimaging study, a vascular study (neurosonological, echocardiographic) was performed, as was an extensive blood test study, with no pathological findings. The radiological evolution of the lesions tended towards resolution, with minimal chronic lesions persisting, compatible with small areas of cerebral malacia (Figure 6).

Thus, in our case, the brain lesions found had the typical radiological features of vasogenic edema. This allows us to exclude, in this case, the ischemic nature of the process (e.g., secondary to arterial occlusion), which would show radiological characteristics of acute ischemic injury (cytotoxic edema) in one or more cerebral vascular territories, as well as a reduction in CBV in MRI perfusion maps. The findings of the radiological evolution contributed to this. Follow-up MRI demonstrated complete resolution of the FLAIR/T2WI signal abnormalities, confirming reversible vasogenic edema, since cytotoxic edema formation and infarction lead to gliotic lesions in the brain and consequently result in permanent signal alterations (hypersignal in long-TR sequences) [24]. Diffusion-weighted MRI reflects the Brownian motion of water molecules within the tissue, thus providing data on tissue integrity. DWI and ADC mapping have mainly been used for the detection of acute ischemia (cytotoxic edema) and for distinguishing between cytotoxic and vasogenic edema [25]. In addition, the ADC map is an MRI image that shows diffusion more accurately than conventional DWI by removing the T2 weighting that is inherent in conventional DWI.

These findings have opened the door to new etiological possibilities for DCS. At this point, in recent years, some cases of breath-hold diving-related DCS have been reported in which, based on neuroimaging findings, an alteration in the permeability of the blood–brain barrier (BBB) has been proposed as a possible underlying pathophysiological mechanism [4,7]. Likewise, some of the neuroimaging findings reported by Matsuo et al. mimicked the features of posterior reversible encephalopathy syndrome (PRES) [4]. These authors proposed two potential pathways by which DCS in breath-hold diving could induce BBB impairment: one is based on mechanical damage and endothelial dysfunction caused by gas bubbles generated by decompression, and the other is that microbubbles or bubble/platelet aggregates in the postcapillary venules cause stasis of capillary flow, leading to BBB impairment [4]. Moreover, dysbaric modification of the BBB has been reported by several researchers previously, in different animal models, using several tracers and under different experimental conditions [26,27,28,29]. Furthermore, it is now known that vascular endothelial cells are vulnerable to diving-related decompression. Thus, it has recently been proposed that circulating bubbles, oxidative stress and other diving-induced stresses are involved in the pathogenesis of endothelial dysfunction [30,31,32,33,34].

These approaches involving endothelial and BBB dysfunction are of recent interest, having been proposed as a key element in the pathophysiology of other clinico-radiological entities such as PRES [35]. Traditionally, the theory proposed for the pathophysiology of PRES was that rapid increases in blood pressures may exceed and overcome the autoregulatory capabilities of the cerebral vasculature, causing vascular leakage and resultant vasogenic edema [35,36]. However, we now know that up to 30% of patients with PRES do not have the high blood pressures necessary to exceed the autoregulatory control of the cerebral vasculature. Here, endothelial dysfunction would be the main culprit, which in turn could be caused by various toxins, immunosuppressive drugs, chemotherapy or septic conditions, among others [35,36,37,38,39]. In PRES, T2WI and T2 FLAIR sequences are probably the most useful, given their sensitivity for detecting vasogenic edema on MRI, as well as DWI sequences and the ADC map. Although the lesions most frequently described in PRES have a bihemispheric distribution and usually involve the parieto-occipital lobes, other locations have been described, with areas such as the frontal and temporal lobes being more common (up to 68% and 40%, respectively) [35,36]. Regarding the radiological evolution of the lesions, PRES-related findings are usually reversible, with normalization of clinical and imaging findings once the underlying etiological cause is resolved. However, in some cases, areas of restricted diffusion may eventually lead to permanent lesions in the brain parenchyma [35]. In this case, ADC map images could have prognostic relevance, since higher values have been associated with lesion reversibility; in contrast, attenuated ADC values would indicate cerebral ischemia and poor neurological outcome [36,37]. The increase in corrected CBV in MRI perfusion would also support the PRES hypothesis with respect to the ischemic mechanism (stroke-like), where it would be decreased. On this last point, there is a potential influence of the increase in cerebral blood flow associated with hypoxemia, with different studies currently showing an increase in blood flow in the middle cerebral artery possibly due to the increase in arterial PaCO_2_ or hypoxia, among others [40,41,42,43].

Finally, in our case, control MRI (1 and 4 months after DCS) demonstrated resolution of the hyperintense bihemispheric vasogenic edema lesions on T2WI/FLAIR sequences and the ADC map, with only minimal areas of cerebral malacia persisting.

## 4. Conclusions

The pathophysiology of DCS in breath-hold diving remains currently unknown. Here, we report a case of DCS in a breath-hold diver. The radiological findings observed in our case, with bihemispheric lesions, hyperintensity in long-TR sequences (T2WI, FLAIR) and no restriction in diffusion sequences (vasogenic edema), suggest an alteration of the BBB. These findings, together with the acute clinical presentation, the resolution of lesions in evolutionary radiological controls and the potential involvement of BBB/endothelial dysfunction reported in recent research studies, could suggest a new form of PRES-like presentation of DCS. Despite this and because of the low prevalence of this pathology, a greater number of cases, studied with adequate neuroimaging protocols, are necessary to deepen our understanding of the pathophysiological mechanisms of neurological breath-hold diving-related DCS.

## Data Availability

Not applicable.
